# MLVA distribution characteristics of *Yersinia pestis *in China and the correlation analysis

**DOI:** 10.1186/1471-2180-9-205

**Published:** 2009-09-23

**Authors:** Xiaoai Zhang, Rong Hai, Jianchun Wei, Zhigang Cui, Enmin Zhang, Zhizhong Song, Dongzheng Yu

**Affiliations:** 1 National Institute for Communicable Disease Control and Prevention, and State Key Laboratory for Infectious Disease Prevention and Control, Chinese Center for Disease Control and Prevention, P. O. Box 5, Changping, Beijing 102206, PR China; 2 Yunnan Institute for Endemic Disease Control and Prevention, Dali 671000, Yunnan, PR China

## Abstract

**Background:**

*Yersinia pestis*, the aetiological agent of plague, has been well defined genotypically on local and worldwide scales. In November 2005, five cases of severe pneumonia of unknown causes, resulting in two deaths, were reported in Yulong, Yunnan province. In this study, we compared *Y. pestis *isolated from the Yulong focus to strains from other areas.

**Results:**

Two hundred and thirteen *Y. pestis *strains collected from different plague foci in China and a live attenuated vaccine strain of *Y. pestis *(EV76) were genotyped using multiple-locus variable-number tandem repeat analysis (MLVA) on 14 loci. A total of 214 *Y. pestis *strains were divided into 85 MLVA types, and Nei's genetic diversity indices of the various loci ranged between 0.02 - 0.76. Minimum spanning tree analysis showed that *Y. pestis *in China could be divided into six complexes. It was observed that Microtus strains were different from the other three biovar strains. Each plague focus had its own unique MLVA types.

**Conclusion:**

The strains isolated from Yulong, Yunnan province had a unique MLVA type, indicating a new clone group. Our results suggest that Yulong strains may have a close relationship with strains from the Qinghai-Tibet Plateau plague focus.

## Background

Plague is an infectious disease caused by *Yersinia pestis*, a naturally occurring bacterium found primarily in wild rodents. It is highly transmissible and brings a high mortality, leading to major public health disasters throughout the history of humanity [1]. In the early 1990s, the incidence of human plague increased significantly [2], with outbreaks occurring in Africa [3] and India [4]. WHO has classified plague as a reemerging infectious disease for the past 20 years, and *Y. pestis *has been identified as a bioterrorism agent, posing as a significant threat to human health and safety [5]. In November 2005, a natural focus of human plague was discovered in Yulong, Yunnan province, China[6]. In this study, we compared *Y. pestis *isolated from the Yulong focus to strains from other areas.

*Y. pestis *couldn't be separated by serotype and phage-type, but could be classified into three biovars: Antiqua, Mediaevalis and Orientalis, according to their ability to ferment glycerol and to reduce nitrate as described by Devignat in the 1950s [7]. Recently, a new biovar Microtus was proposed based on whole genome sequencing and genetic analysis [8,9].

*Y. pestis *has a broad host and vector range [10]. These hosts and vectors have their own natural environment, resulting in the diversity of micro-ecological environments for *Y. pestis*. During its expansion and adaption into new niches, *Y. pestis *undergoes considerable genome variability in response to natural selection. This variability can partly explain the genomic diversity of strains from different plague foci [11]. At present, natural plague foci are widespread inChina. Through systematic analysis of *Y. pestis *in these areas, it is possible to understand the evolution of *Y. pestis *and investigate the source of new plague foci.

Previous studies have revealed a large number of tandem repeat sequences (TRSs) in the *Y. pestis *genome, and these TRSs introduce diversity into various plague strains [12]. These loci are called variable-number tandem repeats (VNTRs). Multiple-locus VNTR analysis (MLVA) is an individual identification method that detects VNTR loci. MLVA is widely used in *Y. pestis *genotyping, and is useful for performing phylogenetic analysis [12-16]. In this study, 213 *Y. pestis *strains collected from different plague foci in China and a live attenuated vaccine strain of *Y. pestis *(EV76) were genotyped by MLVA using 14 loci.

## Methods

### Bacterial strains and DNA preparation

A total of 214 *Y. pestis *strains were included in this study. 208 strains were isolated from 13 natural plague foci in China between 1952 and 2002, an additional five strains were isolated from Yulong Yunnan in 2006, and the EV76 strain was also included in this study (Table 1). The bacteria were cultivated in Hottinger's medium at 28°C for 24 - 36 h, and then the genome DNAs were extracted by using conventional SDS lysis and phenol-chloroform extraction method. The bacterial culture and extraction of DNAs were performed in biosafety level 3 (BSL-3) laboratories.

**Table 1:** The 213 *Y. pestis *isolates used in this study

Plague focus in China	Focus designation in this study		Geographical origin	Year	No. of isolates tested
*Marmota caudate *Plague Focus of the Pamirs Plateau	A		Xinjiang	1956-1997	10
*Marmota baibacina-Spermophilus undulates *Plague Focus of the Tianshan Mountains	B	B1	Xinjiang	------	0
		B2	Xinjiang	1958-1998	12
		B3	Xinjiang	1956-1994	20
		B4	Xinjiang	1975-1987	6
*Marmota himalayana *Plague Focus of the Qinghai-Gansu-Tibet Grassland	C		Tibet, Qinghai, Gansu	1954-1997	38
*Marmota himalayana *Plague Focus of the Qilian Mountain	D		Qinghai, Gansu	1958-2001	20
*Apodemus chevrieri-Eothenomys miletus *Plague Focus of the highland of Northwestern Yunnan Province	E		Yunnan	1954-1994	12
*Rattus flavipectus *Plague Focus of the Yunnan-Guangdong-Fujian provinces	F		Yunnan, Guizhou	1952-2002	22
*Marmota himalayana *Plague Focus of the Gangdisi Mountains	G		Tibet	1966-1998	13
*Spermophilus dauricus *Plague Focus of the Song-Liao Plain	H		Inner Mongolia, Jilin	1953-1970	10
*Meriones unguiculatus *Plague Focus of the Inner Mogolian Plateau	I		Inner Mongolia, Hebei	1970-1995	8
*Spermophilus dauricus alaschanicus *Plague Focus of the Loess Plateau in Gansu and Ningxia provinces	J		Ningxia, Gansu	1962-1978	9
*Marmota himalayana *Plague Focus of the Kunlun Mountains	K	K1	Xinjiang	1972-1979	6
		K2		1985	2
*Microtus brandti *Plague Focus of the Xilin Gol Grassland	L		Inner Mongolia	1970-1987	9
*Microtus fuscus *Plague Focus of the Qinghai-Tibet Plateau	M		Qinghai, Sichuan	1997-2001	10
*Marmota sibirica *Plague Focus of the Hulun Buir Plateau of Inner Mongolia	N		Inner Mongolia	------	0
*Rhombomys opimus *Plague Focus of the Junggar Basin of Xinjiang	O		Xinjiang	------	0
Yulong, Yunnan	P		Yunnan	2006	5

### VNTR locus selection

A total of 14 VNTR loci with core sequences >9 bp were selected from previously described VNTR loci [12,17] (Table 2). The 14 VNTR loci had shown at least two alleles in six sequenced strains of *Y. pestis *(CO92, KIM, 91001, Nepal516, Antiqua, Angola). In order to provide an assay that is useful and widely accessible to research and public health laboratories, the present investigation favors markers with relatively large repeat units.

**Table 2:** VNTR loci and primers used for their amplification ^a^

Locus	Core sequence	Repeat unit size (bp)	Forward and Reverse primer (5'-3')
M76	TGTGGTGATCTAAGCGCAACACA GTTAAAAACAGATTTTAA	41	F:GCGGCCTGATAAGGGATATTGGAAGC R:GGCGAAATTCATTAAAGAGGATCCTGACAC
M73	AATGCCTATTCCTCTGACAGAAT CCGTATC	30	F:GCTTTCTGGCAATGCGATAGTTAGGCATCTC R:GTTAATTTAACTCAATATTGTCGCTATGGT
M72	TTCATAGCTTCTATTATAGAGA	22	F:GCGACACGCCCTTTCAATGAGATACAC R:GTAGATCACCGCTAAATGCGAAGGTCCAC
M66	ATTTACGGTATAGCTACTGA	20	F:GAGATGGATTAACCAGATGTCTTAAAAACTATCGTAAC R:GCGAATCGGCGGCCCAAAC
M61	TTTCAAGCTGAATGTGTG	18	F:GCGCCACAATTAGGGCAACTGC R:GCCGCTTTAATGGTTTGTGAAATGAC
ms01	TGCAGTGAAAAGGTTAAC	18	F:CTAAGCACAATTGTTATGCTGAACC R:TACTGAATCTGCTTCATTGTTCAAA
M59	TTACTGATATGGGCTAG	17	F:GCTTAGCCGCCAGAAAAGGTGAGTTGGC R:GATAATGGCGGTAGCCGGAATCTGATAATCATC
M58	CATAGATAGCTAAACAA	17	F:GCGATAACCCACATTATCACAATAACCAACAC R:GCTGATGGAACCGGTATGCTGAATTTGC
M55	TATTGAGGAATGAATG	16	F:GTCATGGGTGATGCTGTTGCTCTCATTTTATAGTTGTAGTGA R:GCCTTAATGGTTGAATGCGCGAATGAGTCAGATAAC
M54	TTTATGGTTCAACTTG	16	F:GTATGCTTAGCGCCAGTGATAACGAGTC R:GATCGCGTCATCGGGGTTTGTC
M52	GTTGTAAAACCAGAT	15	F:GTGGCCTAACCCGTTTTACCGGTGTAGC R:GCGGTTTTGTCAATCACGAATCAGGACTC
M51	TTATCACGGTTGGTG	15	F:GCAACCCGCTGAAGTTGTAAAAACCGAC R:GCGTTGATCTTCGCGGCCTTCAC
M49	TTGTAACGACTGAT	14	F:GTAATACTTACGCCTTGGCAGCAGTGTTCACGAC R:GTGGGGTGTTCTACGGTGGATTGTTTTTAGGC
M37	ACTATCACCG	10	F:GCCACAGGAAGAGGACATTTCAGAGAAAAC R:GTTGCTAAAACGATACCGCTACGATCAGC

^a^All the primers used here were described in reference 12, except ms01, which was from reference 17.

### VNTR analysis

MLVA was performed as previously described [12]. The loci and corresponding PCR primers were listed in Table 2. PCR reactions were prepared in a 20 μl volume with 10× PCR buffer, 0.5 U *Taq *polymerase, 200 μM each of the four dNTPs, 10 μM each primer set, 1 ng template DNA and filtered sterile water. PCR conditions were as follows: initial denaturation at 94°C for 5 min, and then 35 cycles of 94°C for 20 s, 60°C for 20 s and 72°C for 45 s, followed by a final polymerase extension step at 72°C for 5 min. Strain 91001 was used as a positive control. The PCR products were separated by electrophoresis with a 3% agarose gel (15 cm) in 0.5× Tris-borate-EDTA (TBE) buffer for about 11 h (4 v/cm), and the sizes of amplified DNA fragments were visualized by UV light. Copy numbers were calculated by size and assigned according to the number of repeats for each locus. The PCR products of samples, randomly selected from each number of repeats in each locus, were sequenced and the sequences were compared.

### Data analysis

All data were input into BioNumerics version 4.0 software (Applied Maths, Belgium), cluster analysis and minimal spanning tree (MST) analysis was performed. Nei's indices [18] of VNTR loci and Simpson's diversity index [19] of MLVA typing method were calculated. The formulas were as follows: Nei's index = 1-Σ (allele frequency)^2^; Simpson's diversity index = 1 - Σ [*n*_*j *_(*n*_*j*_-1)]/[*N *(*N*-1)], where N refers to the total number of experimental strains and *n*_*j *_is the number of strains belonging to the *j*th type.

## Results

### Genetic polymorphisms of *Yersinia pestis *in China

**Figure 1:**
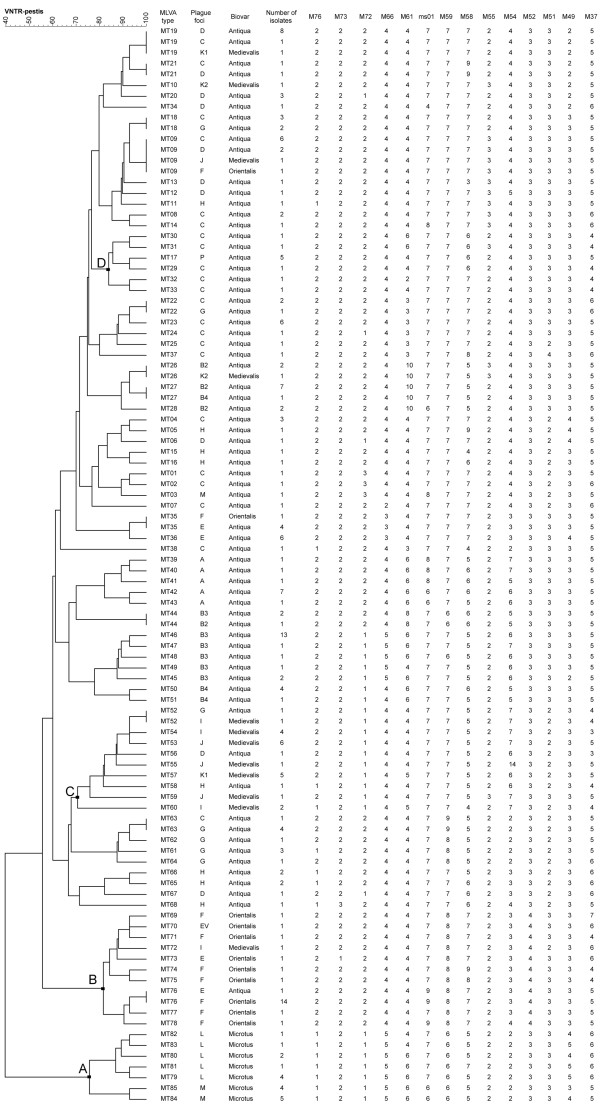
**MLVA genotyping data and cluster analysis**. Cluster analysis was performed using the categorical and unweighted-pair group method using arithmetic averages (UPGMA) options. From left to right, the columns designate the MLVA types, plague foci, biovar, and number of isolates with identical MLVA type and plague focus, repeat number of 14 loci (M76, M73, M72, M66, M61, ms01, M59, M58, M55, M54, M52, M51, M49, and M37). EV (MT70) is strain EV76, which is the vaccine strain. The details of group A, B, C and D were in the text.

Based on analysis of 14 VNTR loci, 214 *Y. pestis *strains were divided into 85 MLVA types (MTs), named as MT01-MT85 (Figure 1). The Simpson's diversity index was 0.9790. The main characteristics of the 14 loci in 214 *Y. pestis *strains were shown in Table 3. Nei's diversity indices of the 14 loci were between 0.02 and 0.76. Locus M54 had higher Nei's diversity index than others. The numbers of alleles of the 14 loci were between 2 and 7. Loci M54, M58 and M61 had the largest number of alleles (n = 7). Locus M58 could further distinguish strains within 15 foci or sub-foci and the other 13 loci could further distinguish strains within 5 to 13 foci or sub-foci. Nei's diversity index of locus M72 was the smallest at 0.02, and it was just able to distinguish strains within two foci (Table 4).

**Table 3:** Main characteristics of the selected VNTR loci in 214 *Y. pestis *strains

Locus	No of alleles	Copy number of repeat sequences	Amplied segment size range	Nei's diversity index
M76	2	1-2	352-393	0.25
M73	3	1-3	319-379	0.02
M72	3	1-3	350-394	0.46
M66	4	2-5	375-435	0.37
M61	7	2-6,8,10	302-374,410,446	0.59
ms01	5	4,6-9	156,192-246	0.33
M59	4	6-9	262-313	0.43
M58	7	3-9	327-429	0.65
M55	2	2,3	395,411	0.18
M54	7	2-7,14	293-373,485	0.76
M52	2	3,4	187,202	0.2
M51	3	2-4	262-292	0.37
M49	4	2-5	291-333	0.35
M37	5	3-7	299-339	0.37

**Table 4:** Number of alleles found among strains from different plague foci in 14 VNTR loci

Locus		M76	M73	M72	M66	M61	ms01	M59	M58	M55	M54	M52	M51	M49	M37
A(11)		1	1	1	1	1	2	1	2	1	3	1	1	1	1
B(38)	B2(12)	1	1	1	1	2	2	2	2	2	2	1	1	1	1
	B3(20)	1	1	2	2	3	1	2	2	1	3	1	1	1	1
	B4(6)	1	1	2	1	2	1	1	2	1	2	1	1	1	1
C(38)		2	1	3	2	4	2	2	6	2	2	1	3	3	3
D(20)		1	1	2	1	1	2	1	5	2	4	1	2	3	3
E(12)		1	2	1	2	1	2	2	1	1	1	2	1	2	2
F(22)		1	1	1	2	1	2	2	3	2	2	2	1	1	3
G(13)		2	1	2	1	2	1	3	2	1	3	1	2	1	3
H(10)		2	2	2	1	1	1	1	5	2	3	1	3	2	3
I(8)		2	1	2	1	2	1	2	3	1	2	2	1	1	3
J(9)		1	1	2	1	1	1	1	2	2	3	1	2	1	1
K(8)	K1(6)	1	1	2	1	2	1	1	2	1	2	1	2	2	1
	K2(2)	1	1	1	1	2	1	1	2	1	1	1	1	2	1
L(9)		1	1	1	1	2	1	1	2	1	1	1	1	2	1
M(10)		2	1	2	2	2	2	2	2	1	2	1	2	2	1
P(5)		1	1	1	1	1	1	1	1	1	1	1	1	1	1

According to cluster analysis based on 85 MTs (Figure 1), Microtus isolates comprising MT79 to MT85 (Group A) could be correctly distinguished from Antiqua, Orientalis and Medievalis. Within Microtus, the strains from two foci (L and M) could be further separated. Most Orientalis isolates were clustered into one major branch (Group B) with the exception of the strains representing MT09 and MT35. Similarly, 80% (20/25) of Medievalis isolates were also clustered together (Group C).

**Figure 2:**
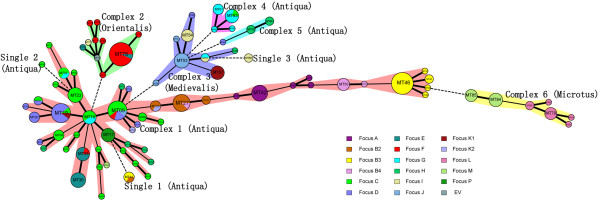
**Minimum spanning tree analysis**. A minimum spanning tree was constructed using the genotyping data provided in figure 1. In the minimum spanning tree the MLVA types are displayed as circles. The size of each circle indicates the number of isolates with this particular type. Thick solid lines connect types that differ in a single VNTR locus and a thin solid connects types that differ in 2 VNTR loci. The colors of the halo surrounding the MLVA types denote types that belong to the same complex. MLVA complexes were assigned if 2 neighboring types did not differ in more than 2 VNTR loci and if at least 3 types fulfilled this criterion.

**Figure 3:**
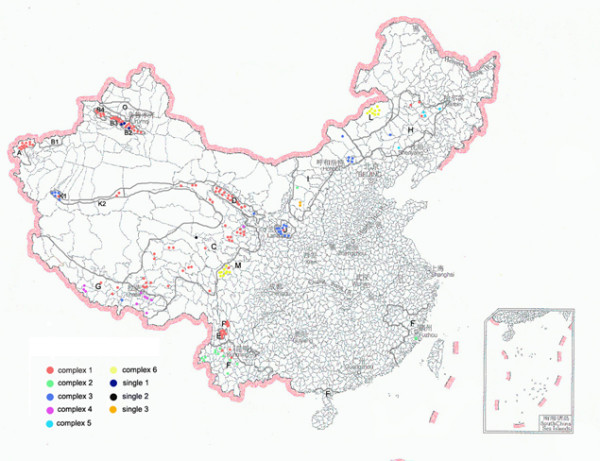
**Distribution complexes in natural plague foci of China**. There are 16 plague foci in China. The names of plague foci represented by letters were according with that in table 1.

As a complementary analysis, a MST analysis was performed based on the categorical data sets (Figure 2). Six complexes and 3 single MTs were obtained. Complex 1, 4 and 5 represented Antiqua isolates and complex 2, 3 and 6 represented Orientalis, Medievalis and Microtus isolates, respectively. Complex 1 contained the largest number of strains (n = 130), which could be divided into 50 MTs. 84.35% (124/147) Antiqua strains were divided into complex 1. It was interesting that the strains isolated from the Xinjiang region (Figure 2, Foci A, B2, B3 and B4) constructed a long branch in complex 1. Complex 2 contained most of the Orientalis isolates, which were all isolated from Focus F (Figure 3). Complex 3 contained 18 Medievalis strains, which was account 72.00% (18/25) of all the Medievalis strains in this study, and three Antiqua strains. Complex 4 and complex 5 were constructed by Antiqua strains. Most of strains in complex 4 were from Focus G, while most of strains in complex 5 were from Focus H. All the Microtus isolates constituted complex 6, which was a well-defined complex representing Microtus isolates.

Strains from each focus presented their own unique MTs. For example, MT39 to MT43 were only found in Focus A, MT44 to MT51 were only found in Focus B, and MT17 was only found in Focus P. A total of 72 MTs were found in the specific foci (Figure 1). However, some strains isolated from different foci could share the same MTs. There were a total of 12 MTs (MT09, 18, 19, 21, 22, 26, 27, 35, 44, 52, 63, and 76) covering strains isolated from different foci. MT09 was shared by 10 strains isolated from 4 foci (C, D, J, F), including the main strains from Focus C. MT19 was shared by 10 isolates from 3 foci (D, C, K), including the main strains from Focus D. The other 10 MTs covered strains of 2 foci.

Most strains from the same focus presented the same or similar MTs (Figure 1). For example, the five strains in Focus P had exactly the same MT (MT17), and 6 of 9 bacteria isolated from Focus J had the same MT (MT53). The 11 strains of Focus A as a subgroup had very similar MTs, including seven strains for MT42 and other four strains for 4 MTs with one to three loci different from MT42. Most of the strains in Focus F were clustered together, including 14 strains for MT76 and the other six strains presenting in 6 MTs. On the other hand, strains from the same focus were dispersed in the cluster tree. For example, strains isolated from Focus G were dispersed in complex 1, 3 and 4, and strains from Focus C were scattered in complex 1 and 4.

### MLVA comparison of *Yersinia pestis *in Yulong and the adjacent foci

Five strains isolated from Yulong, Yunnan had the same MT (MT17: 2-2-2-4-4-7-7-6-2-4-3-3-3-5). Three MTs with a difference in only one locus from MT17 were as follows: MT18 (2-2-2-4-4-7-7-7-2-4-3-3-3-5), including the strains from Foci C and G, had one copy difference on locus M58 with MT17; MT16 (2-2-2-4-4-7-7-6-2-4-3-2-3-5), including a strain which was isolated from Focus H, had one copy difference on locus M51 with MT17; MT29 (2-2-2-4-4-7-7-6-2-4-3-3-3-4), including a strain which was isolated from Focus C, had one copy difference on locus M37 with MT17.

The geographic locations of the natural plague foci adjacent to Yulong were C, E, and F (Figure 3). All the strains from Focus F were Orientalis, and the strains from Foci C, E and Yulong (Focus P) were Antiqua. A further MT comparisons between the Yulong strains and the strains isolated from Foci C and E were as follows: compared with Focus C, It was found that the five Yulong strains and five Focus C strains (belonging to MT29 to MT 33,) were clustered into group D (Figure 1); compared with Focus E, we found one copy difference located at three loci (M66, M58, and M54) in MT35 (major MT) and one copy difference located at four loci (M66, M58, M54, and M49) in MT23 (major MT); The MST analysis (Figure 2) showed that strains from Foci P, C, and E had a close relationship, and almost all strains belonged to one group.

## Discussion

In 2001, Klevytska *et al*. performed a systematic, whole genome analysis of *Y. pestis *CO92, and found that TRSs were widespread and randomly distributed in the bacterial chromosomes and plasmids [12]. Subsequent studies had shown that MLVA could distinguish *Y. pestis *isolated from different natural plague foci [13-15,20]. Our results showed that the loci selected in this study can distinguish the strains from different natural plague foci and even from the same focus. 214 *Y. pestis *strains used in this study were divided into 85 MTs. Simpson's diversity index was 0.9790, indicating that the probability of two unrelated strains being characterized as the same type was 2.10% (1 - 0.9790), showing high resolution and the combination of these 14 loci could be used as a typing method for *Y. pestis *with the generally accepted probability of 5% of type I errors [21].

However, a small number of strains from different foci had the same or similar MTs, suggesting that more VNTR loci should be tried to find out the combination of VNTR loci which could distinguish the strains among different foci completely, or that these strains from different foci may have the same source from another point of view. For example, we observed no considerable differences in the isolation times and places between the only human isolates (N010024, MT03) and the other strains isolated from Focus M. However, we did find a marked difference in MT. In previous studies, epidemiological investigations and traditional ecological typing studies confirmed that this case was imported from Focus C [22,23]. In this study, N010024 was significantly different from the other strains isolated from Focus M, but had very similar MT with the strains from Focus C and gathered with them in the same subgroup. These results coincided with the conclusion of epidemiological investigations and the ecological typing, which further supported MLVA as a bacterial typing method suitable for field epidemiological investigations.

There were cross-types among the MTs of strains from different foci, with MT09 and MT19 being the most prominent. Foci that contained the same MT were geographically close to each other (Figure 3). For example, Foci C, D, F, and J contained MT09, and Foci C, D, and K contained MT19, indicating that there were close relationships among the strains of adjacent foci. It is possible that these strains have the same source. Foci C, D, G, and K have locations adjacent to the border and even similar topography, climate conditions and hosts. The *Marmota Himalayana *plague focus of the Qinghai-Tibet Plateau [24] was sub-divided into four foci in recent years [11]. Cluster analysis showed that majority of the strains in the four foci were in complex 1, indicating a close relationship. Therefore, we suggest that more accurate results will be obtained by combining the four foci in a unit when performing epidemiological and phylogenetic analysis.

Foci A, B, and K are in Xinjiang province (Figure 1). The strains from Foci A and B were in the long branch of complex 1 and obviously different from other strains isolated in China. On the contrary, most strains from Focus K were together with the strains from foci around the Qinghai-Tibet Plateau. Foci A and B are adjacent to the Central Asia foci. Due to the lack of strains outside China in this study, it is impossible to provide a detailed and integrated relationship between the strains in Xinjiang and those of the Central Asia. However, we can confirm that there is a long genetic distance between strains of Foci A, B and other domestic strains isolated in China.

To date, all the strains from Foci L and M belonged to biovar Microtus, except for one imported strain (N010024). Microtus is a newly-identified biovar that is phenotypically and genotypically different from the other three biovars [9]. Our results showed that MLVA could not only differentiate between Microtus and the other three biovars, but also divided the Microtus strains into two subclusters containing strains from foci L and M respectively. Five loci (M61, ms01, M58, M49 and M37) showed differences between the two subclusters. These loci could be used in the subsequent studies focused on Microtus strains.

In November 2005, five cases of severe pneumonia of unknown causes were reported in Yulong, Yunnan province, resulting in two deaths. These cases were subsequently diagnosed as plague, and the natural plague focus was confirmed by field investigation. Five strains of *Y. pestis *were isolated from host animals and vectors. Our results revealed that these five strains had exactly the same MT, suggesting that they had the same source. Furthermore, MT17 was different from the MTs of all the other strains, suggesting that the Yulong strains were a newly-discovered clone. In the 14 selected VNTR loci, M58 was a necessary locus which distinguishes the Yulong strains from the other strains. Moreover, it is also the marker with the second strongest discriminatory ability and the largest number of alleles. Consequently, we propose that M58 is a key locus for MLVA typing of *Y. pestis *in China.

The Yulong focus has distinct geographical features: it is adjacent to Focus E, and both of these foci are in the longitudinal valley area of Western Yunnan, located at the southeast edge of the Qinghai-Tibet Plateau. The two foci are also near Foci C and F. The cluster analysis and MST results suggested that the Yulong strains show a closer genetic relationship with the strains from Focus C than those from Focus E, as is consistent with the results of biological character comparisons [6] and insertion sequence typing [25]. Therefore, it was predicted that the Yulong strains were more likely to be a new branch that evolved from Focus C, rather than the result of expansion and spread of Focus E.

The Yulong natural plague focus is adjacent to the previously-discovered Jianchuan focus (Focus E). Their natural conditions are the same, but the VNTR characteristics of strains from the two foci are critically different, suggesting that the two foci have relatively independent properties because of the hindrance of an ecological barrier. The recent occurrence of "severe pneumonia of unknown causes" in Yulong suggests that plague in this region is a threat to the human population. Since plague has not occurred in the Jianchuan region for a long time, the public health authorities in that area should remain vigilant in monitoring potential plague outbreaks.

## Conclusion

MLVA is a very powerful and reproducible genotyping method and it is promising to be used as a simple molecular tool for characterization and epidemiological studies of *Y. pestis*. It could also unravel the molecular phylogeny of *Y. pestis *when being applied to a larger number of isolates. The 14 loci used in this study gave a high discriminatory power and successfully separated isolates of different biovars and from different natural plague foci. The strains isolated from a new focus, Yulong, Yunnan province had a unique MLVA type, indicating a new clone group. The results of cluster analysis and MST analysis suggest that the Yulong focus strains may have a close relationship with strains from the Qinghai-Tibet Plateau plague focus.

## List of abbreviations

VNTR: variable-number tandem repeat; MLVA: multiple-locus VNTR analysis; TRS: tandem repeat sequences; MT: MLVA type; MST: minimum spanning tree.

## Authors' contributions

ZXA did most of the typing work. WJC prepared the DNA samples. CZG was in charge of the Bionumerics database, clustering analyses and minimum spanning tree analyses. ZEM and SZZ were in charge of the epidemiological investigation and collection of Yulong strains. HR and YDZ initiated and managed the project, and ZXA and HR wrote the report. All authors read and approved the final manuscript.
